# Evaluation of a novel vaginal cells self‐sampling device for human papillomavirus testing in cervical cancer screening: A clinical trial assessing reliability and acceptability

**DOI:** 10.1002/btm2.10653

**Published:** 2024-03-13

**Authors:** Chung‐Yao Yang, Ting‐Chang Chang, Hung‐Hsueh Chou, Angel Chao, Shih‐Tien Hsu, Yu‐Hsiang Shih, Huei‐Jean Huang, Cheng‐Tao Lin, Min‐Yu Chen, Lou Sun, Kuan‐Gen Huang, Kai‐Yun Wu, Wu‐Chiao Hsieh, Yi‐Ting Huang, Liang‐Hsuan Chen, Chien‐Hsing Lu, Hao Lin, Chao‐Min Cheng

**Affiliations:** ^1^ Hygeia Touch Inc. Taipei Taiwan; ^2^ Division of Gynecologic Oncology, Department of Obstetrics and Gynecology Chang Gung Memorial Hospital, Linkou Branch Taoyuan Taiwan; ^3^ Gynecologic Cancer Research Center, Chang Gung Memorial Hospital Taoyuan Taiwan; ^4^ School of Medicine, National Tsing Hua University Hsinchu Taiwan; ^5^ Department of Gynecology and Obstetrics Taichung Veterans General Hospital Taichung Taiwan; ^6^ Department of Obstetrics and Gynecology Kaohsiung Chang Gung Memorial Hospital Kaohsiung Taiwan; ^7^ Institute of Biomedical Engineering, National Tsing Hua University Hsinchu Taiwan

**Keywords:** acceptability, cervical cancer screening, HPV testing, reliability, self‐sampling

## Abstract

Cervical cancer is a significant public health concern, particularly in low‐ and middle‐income countries where resources for prevention and treatment are limited. Routine screening, such as the Papanicolaou test (Pap smears) and human papillomavirus (HPV) testing, plays a crucial role in the early detection and prevention of cervical cancer. However, the participation rate in cervical cancer screening programs remains below optimal levels due to various factors. This study aimed to evaluate the reliability and acceptability of the HygeiaTouch Self Sampling Kit for Women in collecting vaginal samples for HPV typing, comparing the results with samples collected by physicians. The study included 1210 women aged 21–65 from three medical centers in Taiwan. The findings indicated that the self‐sampling kit was as effective as physician‐collected specimens in terms of obtaining valid samples and identifying HPV. The agreement between the two methods was 88%, with a *κ* value of 0.75. Furthermore, the study assessed the mechanical characteristics of the self‐sampling applicator through tensile, bending, and torque tests, and determined that it was safe for intravaginal use. Additionally, the study evaluated the safety and satisfaction of self‐sampling and found a low rate of adverse events (0.7%) and high levels of satisfaction (over 90%) among participants. Overall, we demonstrated that the HygeiaTouch Self Sampling Kit for Women is a reliable and acceptable device for HPV testing and cervical screening, providing a convenient, safe, and effective alternative for women.

**Translational Impact Statement**

Cervical cancer is a global health concern, especially in low‐ and middle‐income countries. Routine screening, including Pap smears and HPV testing, is vital for early detection and prevention. We evaluated the HygeiaTouch Self Sampling Kit, comparing it to physician‐collected samples in a study of 1210 women. Results showed the kit was as effective as physician collection, with an 88% agreement. Mechanical tests confirmed its safety, and participants reported high satisfaction (over 90%). HygeiaTouch is a reliable and convenient device for cervical screening and HPV testing.

## 1 INTRODUCTION

Cervical cancer is a prevalent form of cancer among women worldwide, ranking fourth in terms of frequency. It poses a significant challenge, particularly in low‐ and middle‐income countries where healthcare resources and preventive measures are limited. Early detection plays a crucial role in the prevention and treatment of cervical cancer, making routine screening, such as Papanicolaou test (Pap smears) and human papillomavirus (HPV) testing, essential. Despite the existence of well‐structured national screening programs that are free of charge, the participation rate remains suboptimal, with only about three‐quarters of invited women attending. The participation rate of Taiwanese women aged 30–69 in cervical cancer screening, specifically Pap smears or HPV testing, has consistently been around 30% annually and 69% every 3 years. A significant number of women still do not engage in these screening processes. Our previous research has identified several reasons for their non‐participation,^1^ including feelings of embarrassment (36.4%), lack of time (35.8%), forgetfulness (25.9%), underestimation of personal risk (24.4%), fear of receiving a positive result (13.1%), and concerns about potential pain (10.8%). Therefore, it is crucial to develop and implement novel strategies aimed at enhancing adherence to cervical cancer screening.

Cervical screening using high‐risk HPV (hrHPV) tests has shown greater sensitivity and negative predictive value compared to cytological screening, as supported by multiple studies.^2^, ^3^, ^4^, ^5^, ^6^ An increasing number of countries are adopting self‐sampling as a method for offering cervical screening.^7^ In addition, self‐sampling is now being offered to all women of screening age, rather than just those who are difficult to reach. This shift is supported by evidence suggesting that HPV testing on self‐samples is as effective as testing on samples taken by physicians for detecting high‐grade cervical lesions.^8^ While self‐sampling is an important development in achieving the World Health Organization's goals for eliminating cervical cancer, it is important to note that HPV testing on self‐samples is not as advanced in terms of technical optimization and workflow. This is evident in the limited number of clinically validated HPV assays that have been approved for use with self‐collected samples compared to samples taken by physicians.

In the initial phase, there has been a notable increase in the availability of collection devices for self‐sampling. A review conducted by the National Health Service (NHS, England) approximately a decade ago identified 43 devices that were either specifically designed for self‐sampling or had the potential to be used for this purpose.^9^ At that time, only a few devices had obtained CE‐marking for vaginal collection, namely the Evalyn Brush (Rovers Medical, Oss, The Netherlands), HerSwab (Eve Medical, Toronto, ON, Canada), Delphi Screener (Rovers Medical, Oss, The Netherlands), and Qvintip (Aprovix, Solna, Sweden). Since then, additional devices such as the Copan FLOQSwab (Copan Italia S.p.A., Brescia, Italy) and Rovers Viba Brush (Rovers Medical, Oss, The Netherlands) have also obtained CE‐marking for vaginal collection. To date, several studies have demonstrated that there is a high level of agreement in the detection of high‐risk human papillomavirus (hrHPV) between physician‐sampled cervical specimens and self‐collected vaginal specimens obtained using various collection devices, including FLOQSwabs, Evalyn Brush, and HerSwab.^10^, ^11^, ^12^, ^13^, ^14^

It is important to have a range of well‐validated devices as it would be simplistic to assume that one device would be suitable for all settings. Local decision‐making, taking into account factors such as target population, climate, cultural norms, and existing laboratory and logistical infrastructure, will be crucial. While further data on the long‐term stability and tolerance of HPV deoxyribonucleic acid (DNA) on existing self‐sampling devices would be valuable, there is evidence to suggest that HPV DNA remains analytically stable for up to 32 weeks on certain dry devices.^15^, ^16^ Dry devices are appealing as they eliminate the risk of spillage or leakage. However, samples in a buffer solution may offer the advantage of increased stability of biomaterial or suitability for other tests, such as analysis of the vaginal microbiome. Despite widespread evidence supporting the effectiveness of vaginal self‐sampling for hrHPV detection, there is a growing need to evaluate self‐collection devices in terms of safety, comfort, and ease of use. Furthermore, this field is rapidly evolving, and it is important for the scientific community to stay informed about new evidence in the literature and emerging claims from HPV assay manufacturers. In this study, we conducted a clinical trial to assess the effectiveness of a newly designed self‐collection kit for collecting vaginal samples for HPV typing, comparing the results with those obtained from physician‐sampled cervical specimens. Additionally, the user experience of self‐sampling using the self‐collection kit was evaluated through a questionnaire, while the safety of the self‐collection kit was assessed through a mechanical properties test.

## 2 MATERIALS AND METHODS

### 2.1 Study population

Between June 2020 and November 2021, a total of 1210 women aged between 21 and 65 years were recruited from Linkou Chang Gung Memorial Hospital, Kaohsiung Chang Gung Memorial Hospital, and Taichung Veterans General Hospital for the purpose of this study in Taiwan. Prior to participation, formal consent was obtained from all participants. In order to be included in the study, participants had to meet one of the following criteria: (a) no history or current presence of cervical intraepithelial lesion or malignancy; (b) a history of mildly abnormal or low‐grade Pap test or cervical histology within the past 3–12 months, including atypical squamous cells of undetermined significance (ASCUS), cervical intraepithelial neoplasia grade 1 (CIN1), or atypical glandular cells (AGC); (c) previous treatment for high‐grade cervical lesion(s) or a history of high‐grade cytology, including atypical squamous cells that did not exclude high‐grade squamous intraepithelial lesion (ASC‐H), high‐grade squamous intraepithelial lesion (HSIL), cervical intraepithelial neoplasia grade 2 (CIN2), cervical intraepithelial neoplasia grade 3 (CIN3), cervical carcinoma in situ (CIS), squamous cell carcinoma (SCC), atypical glandular cells favor neoplasm (AGCFN), adenocarcinoma in situ (AIS), or cervical adenocarcinoma; (d) current presence of mildly abnormal or low‐grade Pap test or cervical histology, such as ASCUS, CIN1, or AGC; and (e) current presence of high‐grade cytology or high‐grade cervical lesion(s), including ASC‐H, HSIL, CIN2, CIN3, CIS, SCC, AGCFN, AIS, or cervical adenocarcinoma. Women who had undergone total hysterectomy or had a congenital cervical anomaly, were pregnant at the time of their clinical visit, had cervicitis requiring treatment, had undergone cervical lesion surgery other than biopsy within the past 90 days, or had received or were receiving radiotherapy targeting the uterus, cervix, or vagina, were excluded from the study. Additionally, women who had engaged in sexual activity without using a condom within the past 48 h, had excessive vaginal discharge due to ovulation or inflammation, had an intravaginal tablet or residual medication in the vaginal canal, or were menstruating, were allowed to participate in the study following the resolution of these factors.

This study will utilize the test outcomes of specimens collected by physicians as the standard and compare them with the HPV type test outcomes for self‐collected specimens from the same participant to validate the consistency of HPV type between the two sampling methods. The study's design was structured based on the stratification of participants according to their cervical precancerous lesion status. The intended enrollment figures for the respective groups were 120 for criteria a (with an expected 10% HPV positivity), 180 for criteria b (anticipated 70% positivity), 240 for criteria c, 240 for criteria d, and 420 for criteria e, with estimated HPV positivity rates of 50%, 70%, and 90%, respectively. The total projected enrollment was established at 1200 participants, with an overall anticipated HPV positivity rate of 67%. Ethical approval for this study was obtained from the Institutional Review Boards of Chang Gung Medical Foundation (IRB No. 20200618A0) and Taichung Veterans General Hospital (IRB No. SF20101B).

### 2.2 Sample collection and kits

**FIGURE 1:**
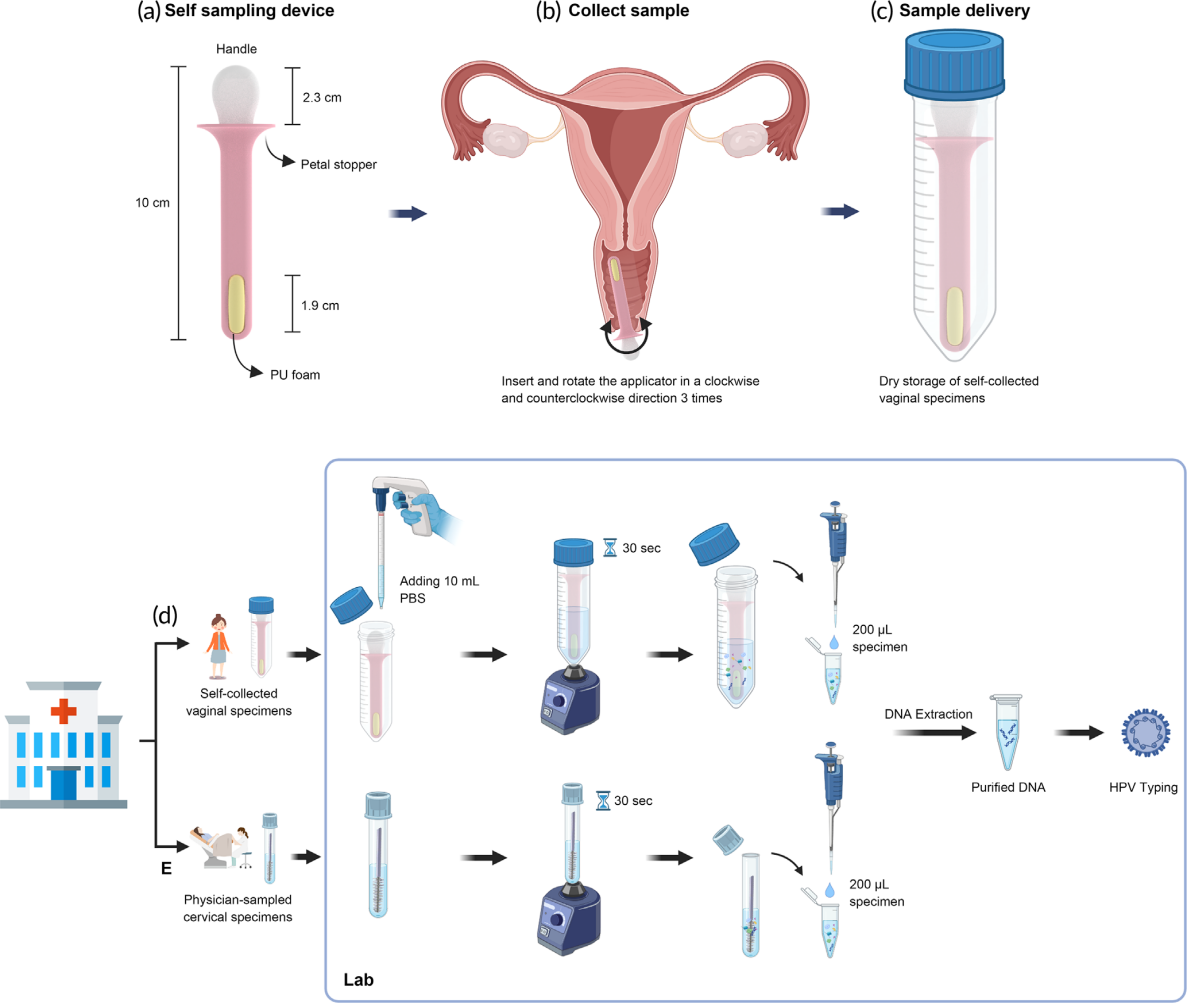
Schematic diagrams of various components and procedures related to the self‐sampling device and sample processing procedures in this study. (a) The self‐sampling device provided to participants for the study is approximately 10 cm long, with a comfortable grip handle measuring 2.3 cm and a polyurethane foam sample collection pad embedded in the distal end. (b) Participants were provided instructions on how to perform the self‐collection procedure during their visit to the outpatient clinic. (c) Specimens were stored in a sterilized 50 mL centrifuge tube in a dry state for transportation to the laboratory for further processing and analysis. (d) Initially, a self‐collected vaginal specimen was mixed with 10 mL of PBS buffer and then vortexed for 30 s to wash cells from the foam. From this, 200 μL of the specimen was transferred into a microtube and subjected to DNA extraction protocol. (e) In the case of physician‐sampled cervical specimens, the cells from the brush were washed by vortexing for 30 s. From this, 200 μL of the specimen was then transferred into a microtube and subjected to the DNA extraction protocol. Both purified DNA samples were subjected to HPV genotyping using the DR. HPV Genotyping IVD Kit, following the manufacturer's instructions. The diagram was created using BioRender.com (https://app.biorender.com/).

The HygeiaTouch Self Sampling Kit for Women, developed by Hygeia Touch Inc. in Taipei, Taiwan, is designed to facilitate the collection of exfoliated cells from the vaginal fornix. This kit utilizes a sterile vaginal applicator that features a biocompatible, thermoplastic rubber‐coated, polypropylene stick with an embedded, highly absorbent, soft foam collection pad at the distal end. This collection pad, constructed of soft polyurethane (PU) measuring 19 × 5 mm serves as the cell sample collection surface (Figure 1a). The device also features a petal stopper, which allows for safe control of insertion depth. The primary objective of this applicator is to offer adult women a comfortable and convenient means of self‐sample collection.

Upon obtaining informed consent, participants were furnished with comprehensive instructional materials, consisting of a manual and a brief video, elucidating the proper technique for self‐collecting vaginal specimens, and a complete sampling kit. After inserting and rotating the applicator clockwise and counterclockwise three times, the applicator was removed from vagina and placed into a sample‐collection tube (50 mL centrifuge tube) without preservation medium (Figure 1b,c). The tube was then given to the study nurse and sent to a laboratory at room temperature within 48 h. After finishing self‐sampling, the participant was asked to answer the satisfaction questionnaire.

Upon the completion of the questionnaire, participants proceeded to the outpatient clinic where they underwent specimen sampling and received follow‐up medical treatments administered by the attending physician. The physician utilized a LIBO Cytology Brush Kit (Iron Will Biomedical Technology, New Taipei, Taiwan) to obtain a cytology sample from both the cervical surface and endocervical canal. The collected sample was subsequently dispersed into 2 mL phosphate buffered saline (PBS) buffer. Following sample collection, colposcopy, cervical biopsy, or other relevant diagnostic procedures were performed in accordance with the World Health Organization's guidelines for the management of cervical pre‐cancerous lesions.^17^ Within 48 h, the sample was then transported to the laboratory at room temperature.

The tube used for sample collection was pre‐coded using a sticker. The code assigned to the specimen collected by the physician was determined based on the study group and the order of entry. The self‐collected specimen was coded using a random number generated in SAS 9.4 (SAS Institute Inc., Cary, NC, USA). No personal patient information was included on the tube. The laboratory technician was blinded in the pairing of specimens.

### 2.3 Sample processing and HPV screening

The self‐collected specimen was rinsed from the applicator after adding 10 mL of PBS buffer into the sample‐collection tube and vortexing for 30 s (Figure 1d). The physician‐sampled specimen was rinsed from the brush after vortexing for 30 s (Figure 1e). Genomic DNA was extracted from each sample (200 μL) using the QIAGEN QIAamp DNA Mini Kit (Qiagen, Hilden, Germany). The purified DNA (20 ng) was stored at −20°C until subsequent HPV genotyping. HPV typing for both the physician‐sampled specimen and self‐collected specimen was performed using the DR. HPV Genotyping IVD Kit (DR. Chip Biotech, Inc., Miaoli, Taiwan) was used according to the manufacturer's instructions at the laboratory of DR. Chip Biotech, Inc. This kit is capable of detecting a total of 27 HPV types, including types 6, 11, 16, 18, 31, 33, 35, 39, 45, 51, 52, 53, 54, 56, 58, 59, 61, 62, 66, 68, 69, 70, 72, 73, 81, 82, and 84. A specific gene fragment from HPV was amplified by PCR using the HEMA 9700 gene amplifier (Zhuhai Hema Medical Instrument Co., Ltd., Zhuhai, China) under the following conditions: 95°C for 10 min, 35 cycles of 95°C for 30 s, 50°C for 30 s, and 72°C for 50 s, followed by a final elongation step of 72°C for 7 min and preservation at 4°C. Three microliters of the resulting amplimers were hybridized with an oligonucleotide array that was pre‐spotted on a plastic chip at 50°C for 40 min using the DR. Mini Oven (DR. Chip Biotech, Inc., Miaoli, Taiwan). After the hybridization reaction, the hybridized target DNA was detected through enzymatic, colorimetric development, and the signal was captured and analyzed using DR. Chip's imaging device—DR. AiM Reader (DR. Chip Biotech, Inc., Miaoli, Taiwan).^18^

### 2.4 Mechanical properties test

**FIGURE 2:**
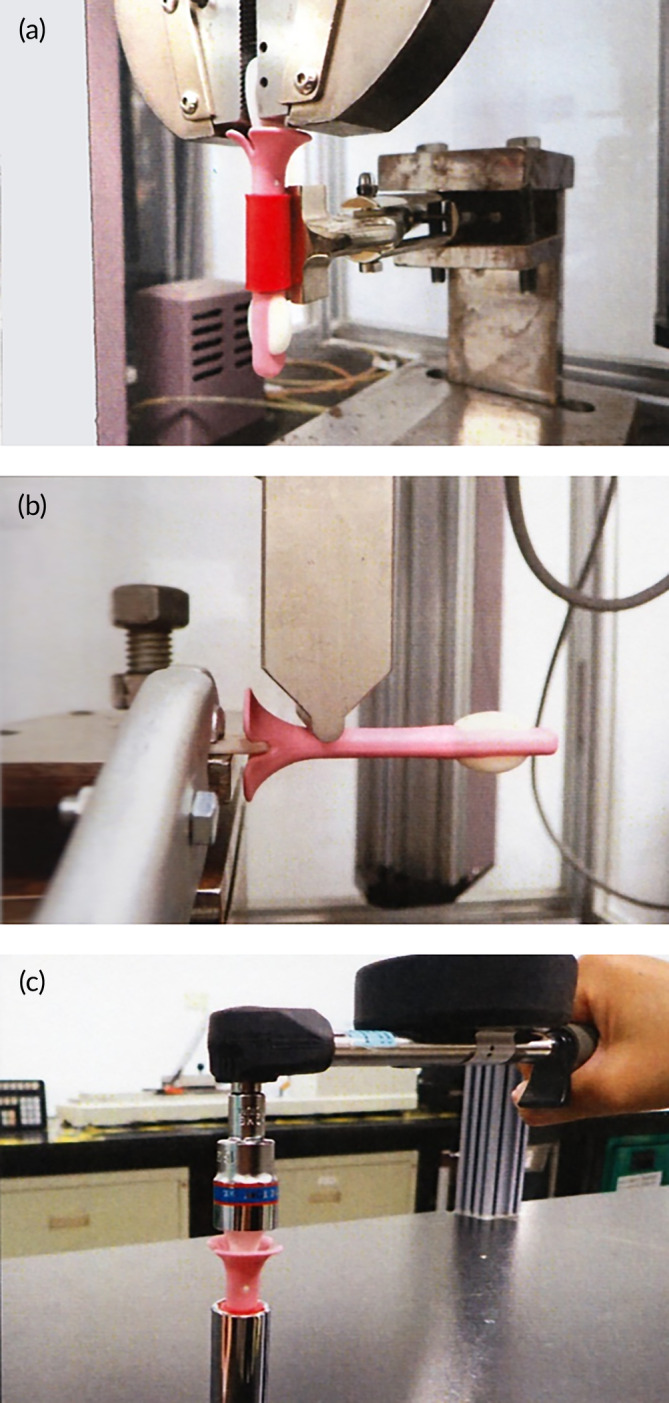
Diagrams illustrating the mechanical properties evaluation of a self‐sampling applicator are presented in this study. The diagrams depict the results of three distinct tests: (a) tensile testing, (b) bending testing, and (c) torque testing.

The mechanical properties of the applicator were assessed through a series of tests including tensile, bending, and torsion evaluations. Prior to conducting the tests, the applicators were stored in a controlled environment at a temperature of 23°C (±2°C) and a relative humidity of 50% (±5%) for a minimum of 24 h. Each test was carried out using five samples, all subjected to the same environmental conditions. Tensile testing was performed using MTS C43.104 (MTS Systems, Eden Prairie, MN, USA), with a testing speed of 50 mm/min, as depicted in Figure 2a. The bending test was also conducted using MTS C43.104, with a test acceleration speed of 10 mm/min, as shown in Figure 2b. The torsion test was conducted using a Tohnichi 15DB4 apparatus (Tohnichi Mfg. Co., Ltd., Tokyo, Japan), with a twist angle of 90°, and the maximum torque value was recorded, as illustrated in Figure 2c.

In relation to the standards for tensile strength, bending strength, and torque testing of the self‐sampling applicator, it is estimated that the force applied by the user within the vaginal cavity will not surpass 2 kgf. This amount is equivalent to the force required to horizontally lift six 300 mL aluminum foil packaged beverages (approximately 1.8 kg) by hand. Exerting a force greater than 2 kgf on the vaginal wall may result in discomfort for the user. Consequently, the acceptance specification for the mechanical test was established at 2 kgf. If the mechanical test yields a result exceeding this threshold, it suggests that the chances of a user damaging the self‐sampling applicator and inadvertently retaining it within the vagina is very low.

### 2.5 Data analysis

The concordance between the self‐collected and physician‐sampled specimens in terms of HPV types was assessed using Cohen's kappa coefficient (*κ*). The coefficient ranges were categorized as follows: slight agreement (0–0.20), fair agreement (0.21–0.40), moderate agreement (0.41–0.60), substantial agreement (0.61–0.80), and almost perfect agreement (0.81–1).^19^ The overall agreement percentage between the paired samples was determined by dividing the number of concordant sample sets by the total number of samples. In cases where multiple histological results were available, the most severe diagnosis was considered. Statistical analyses were conducted using SAS software.

## 3 RESULTS

### 3.1 Participants demographics

Between the dates of May 29, 2020, and November 19, 2021, a total of 1210 participants were enrolled in the study. All of the enrolled participants were included in the safety assessment. Forty cases were excluded from the evaluation of HPV type agreement. This exclusion consisted of 13 cases that did not meet the inclusion criteria and 27 cases that were enrolled prior to the acceptance of the formal letter of study agreement from one of the participating institutions. Table 1 presents an overview of the fundamental characteristics of the study population. The study included 1170 women, ranging in age from 21 to 65 years old, with an average age of 45.3 years (standard deviation, SD = 10.2). Approximately 29.8% of the participants were in menopause. From the participants who underwent histological examination, 52.6% (347 out of 660) were found to have CIN2 or worse (CIN2+). Among all participants, the age group of 21–29 demonstrated the highest percentage (60%) of individuals with CIN2+, which represented 27 out of 45 participants. Following closely behind were those aged 30–39 (57.4%), or 108 out of 188 participants. However, when considering the proportion of participants diagnosed with cancer, a contrasting trend emerges. The highest proportion of cancer cases was found among participants aged 60–65, with 20.9% (14 out of 67) of participants in this age group receiving cancer diagnoses. This is followed by the 50–59 age group, where 13.4% (21 out of 157) of participants were diagnosed with cancer.

**TABLE 1:** Key demographic information of participants involved in the assessment of agreement in HPV sampling.

Characteristics	Age group (year)					
	21–29 (*N* = 66)	30–39 (*N* = 301)	40–49 (*N* = 383)	50–59 (*N* = 304)	60–65 (*N* = 116)	Total (*N* = 1170)
Age (year)						
Mean ± SD	26.5 ± 2.2	35.2 ± 2.9	44.2 ± 2.9	54.3 ± 2.8	62.4 ± 1.9	45.3 ± 10.2
Menopause, *n* (%)						
No	66 (100)	299 (99.3)	364 (95.0)	92 (30.3)	0 (0)	821 (70.2)
Yes	0	2 (0.7)	19 (5.0)	212 (69.7)	116 (100)	349 (29.8)
HPV^a^, *n* (%)						
Positive	53 (80.3)	212 (70.4)	222 (58.0)	155 (51.0)	78 (67.2)	720 (61.5)
Negative	13 (19.7)	89 (29.6)	161 (42.0)	149 (49.0)	38 (32.8)	450 (38.5)
Current histology^b^, *n* (%)						
Biopsy	45 (68.2)	188 (62.5)	203 (53.0)	157 (51.6)	67 (57.8)	660 (56.4)
Condyloma	1 (1.5)	1 (0.3)	4 (1.0)	2 (0.7)	2 (1.7)	10 (0.9)
CIN1	11 (16.7)	42 (14.0)	43 (11.2)	49 (16.1)	15 (12.9)	160 (13.7)
CIN2	11 (16.7)	42 (14.0)	37 (9.7)	16 (5.3)	15 (12.9)	121 (10.3)
CIN3	13 (19.7)	54 (17.9)	52 (13.6)	31 (10.2)	7 (6.0)	157 (13.4)
Invasive cancer	3 (4.5)	12 (4.0)	19 (5.0)	21 (6.9)	14 (12.1)	69 (5.9)
CIN/malignancy (−)	6 (9.1)	37 (12.3)	48 (12.5)	38 (12.5)	14 (12.1)	143 (12.2)
No biopsy	21 (31.8)	113 (37.5)	180 (47.0)	147 (48.4)	49 (42.2)	510 (43.6)

*Abbreviation*: CIN, cervical intraepithelial neoplasia.

^a^ The test results of physician‐sampled specimens.

^b^ Histology at enrollment.

### 3.2 HPV results of self‐collected and physician‐sampled specimens

The self‐sampling kit demonstrated comparable efficacy to the physician‐sampled method in terms of obtaining valid specimens, with both methods achieving a 100% success rate. In relation to the identification of HPV, it was found that 61.5% (720 out of 1170) of specimens collected by physicians and 61.4% (718 out of 1170) of self‐collected specimens yielded positive results for HPV. The level of concordance between the two methods was determined to be 88%, with a *κ* value of 0.75 (Table 2). A total of 720 cases were found to have positive HPV in physician‐sampled specimens. Among these cases, 69% (497 out of 720) were identified as single‐type infections, while 31% (223 out of 720) were classified as multiple‐type infections. Similarly, in the self‐collected specimens, 66.2% (475 out of 718) were single‐type infections and 33.8% (243 out of 718) were multiple‐type infections. The study assessed the concordance rates between HPV detection results in self‐collected specimens and physician‐sampled specimens, finding that the percent positive agreement was 90.1% (649 out of 720) and the percent negative agreement was 84.7% (381 out of 450). The agreement between the two methods for detecting the most common high‐risk HPV (hrHPV) types (16, 18, 31, 33, 35, 39, 45, 51, 52, 56, 58, 59, 66, and 68) was found to be 87.7%, with a *κ* value of 0.75. For HPV 16/18, the level of concordance was determined to be 94.6%, with a value of 0.80.

**TABLE 2:** Agreement of HPV detected between self‐collected specimens and physician‐sampled specimens.

Self‐collected	Physician‐sampled		Kappa statistic (95% CI)	*p* Value^a^	Agreement^b^ (%) (95% CI)
	Positive	Negative			
Any HPV^c^					
Positive	649	69	0.75 (0.71–0.79)	<0.0001	88.0 (86.2–89.9)
Negative	71	381			
hrHPV^d^					
Positive	524	57	0.75 (0.72–0.79)	<0.0001	87.7 (85.8–89.6)
Negative	87	502			
HPV 16/18^e^					
Positive	156	20	0.80 (0.75–0.85)	<0.0001	94.6 (93.3–95.9)
Negative	43	951			

^a^ Pearson's chi‐square test was used to test for differences between two methods.

^b^ Defined as either (1) presence of any HPV type in physician‐sampled specimen and self‐collected specimen or (2) absence of HPV in physician‐sampled specimen and self‐collected specimen.

^c^ Any HPV: HPV 6, 11, 16, 18, 31, 33, 35, 39, 45, 51, 52, 53, 54, 56, 58, 59, 61, 62, 66, 68, 69, 70, 72, 73, 81, 82, and 84.

^d^ hrHPV: high risk HPV including HPV 16, 18, 31, 33, 35, 39, 45, 51, 52, 56, 58, 59, 66, and 68.

^e^ HPV type 16 or 18.

Table 3 presents the clinical performance for detecting CIN2+ cases. Out of 347 identified CIN2+ cases, physician‐sampled specimens missed 40, while self‐collected specimens missed 56. Consequently, the sensitivity and specificity of physician‐sampled specimens for CIN2+ detection were 88.5% and 49.8%, respectively, while self‐collected specimens exhibited a sensitivity of 83.9% and specificity of 48.1%. The effectiveness of HPV for identifying CIN2+ via self‐collected specimens closely mirrors that of physician‐sampled specimens, with a relative accuracy of 0.96 (0.90–1.03).

**TABLE 3:** Clinical performance of HPV tests against CIN2+ detection of two sampling methods (*N* = 1170).

	*N*	CIN2+	Sensitivity (%, 95% CI)	Specificity (%, 95% CI)	PPV (%, 95% CI)	NPV (%, 95% CI)	AUC
Physician‐sampled							
Positive	720	307	88.5 (85.7, 92.5)	49.8 (46.4, 53.2)	41.6 (38.0, 45.2)	91.9 (89.4, 94.5)	0.691
Negative	450	40					
Self‐collected							
Positive	718	291	83.9 (80.6, 88.4)	48.1 (44.7, 51.5)	39.5 (35.9, 43.1)	88.6 (85.6, 91.5)	0.660
Negative	452	56					

*Abbreviations*: AUC, area under ROC curve; CI, confidence interval; NPV, negative predictive value; PPV, positive predictive value.

**FIGURE 3:**
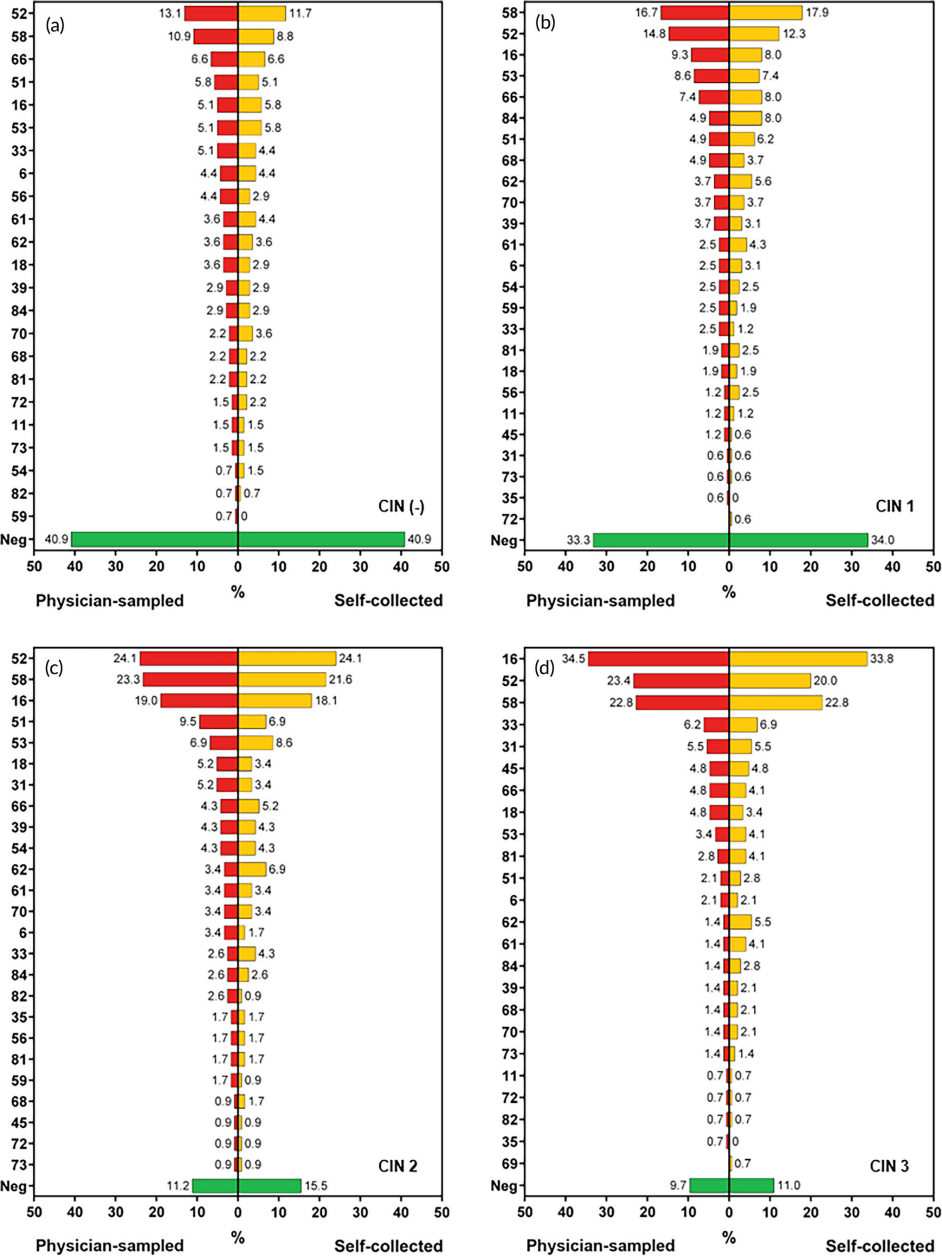
The prevalence of different HPV types in physician‐sampled specimens and self‐collected specimens, categorized by different histological statuses: (a) no precancerous lesions or malignancy (*n* = 143), (b) CIN1 (*n* = 160), (c) CIN2 (*n* = 121), and (d) CIN3 (*n* = 157).

Figure 3 illustrates the distribution of HPV types in physician‐sampled specimens and self‐collected specimens, categorized according to different histological findings such as CIN1, CIN2, CIN3, and those without precancerous lesions or malignancy. Notably, HPV 52 and HPV 58 were prevalent types across all groups. However, it is worth noting that among individuals diagnosed with CIN3, a substantial proportion (34%) tested positive for HPV 16. This finding underscores the significance of HPV 16 as not only the most pertinent type for cervical cancer but also the most prevalent type associated with CIN3.

### 3.3 Mechanical characteristics of the self‐sampling applicator

Table 4 displays the mechanical characteristics of the HygeiaTouch self‐sampling applicator at its maximum load. These properties include the tensile strength, bending strength, and torque, which measure at 3 kgf (SD = 0.2), 5.8 kgf (SD = 0.7), and 3.5 kgf cm (SD = 0.1), respectively. Based on the aforementioned three tests, the results surpassed the initial evaluation of 2 kgf. Specifically, the self‐sampling applicator exhibited a bending resistance strength of 5.8 kgf, which is roughly equivalent to the force needed to horizontally lift approximately 10 bottles of 600 mL PET bottles with one hand. It is anticipated that users will not exert such significant force within the vaginal area, thus the likelihood of the self‐sampling applicator deforming or fracturing in this context is minimal. Consequently, the HygeiaTouch self‐sampling applicator has been deemed safe for intravaginal use.

**TABLE 4:** Mechanical characteristics of the self‐sampling applicator via tensile, bending, and torque testing.

Items		Results
Tensile test		Tensile at max load (kgf)
	#1	3.3
	#2	2.8
	#3	2.9
	#4	3.2
	#5	3.0
	Mean (SD)	3.0 (0.2)
Bending test		Bending at max load (kgf)
	#1	5.1
	#2	5.2
	#3	6.3
	#4	5.6
	#5	6.7
	Mean (SD)	5.8 (0.7)
Torque test		Torque at max load (kgf cm)
	#1	3.4
	#2	3.4
	#3	3.6
	#4	3.6
	#5	3.6
	Mean (SD)	3.5 (0.1)

### 3.4 Assessment of safety and self‐sampling satisfaction

During the course of this trial, a total of nine participants experienced mild adverse events while engaging in self‐sampling. Specifically, two participants reported experiencing mild anxiety, as classified by the Common Terminology Criteria for Adverse Events (CTCAE) as Grade 1 anxiety. Additionally, seven participants reported experiencing mild vaginal pain, classified as CTCAE Grade 1 perineal pain. It is important to note that these adverse events occurred immediately during the self‐sampling process and ceased once the sampling was completed, without any further continuation. The overall rate of adverse reactions was determined to be 0.7% (9 out of 1210). The findings from the questionnaire survey in Table 5 revealed that a significant majority of participants, specifically 99.3%, believed that the self‐sampling process was safe. Furthermore, 94.8% of participants found the self‐sampling process to be easy. Additionally, a substantial proportion of participants, 90.6%, expressed their willingness to utilize self‐sampling for cervical screening. Numerous factors contribute to feelings of discomfort, primarily arising from vaginal dryness and challenges associated with insertion. The demographic most affected by these concerns comprises women who have undergone menopause.

**TABLE 5:** Satisfaction survey of HPV self‐sampling test using “HygeiaTouch Self Sampling Kit for Women” (*N* = 1210, ITT^a^).

Question	Not at all		Low		High		Extremely	
	*N*	%	*N*	%	*N*	%	*N*	%
1. Satisfaction level of the design of the self‐sampling kit	5	(0.4)	32	(2.6)	896	(74.0)	277	(22.9)
2. User‐friendly level of using the self‐sampling kit	2	(0.2)	37	(3.1)	594	(49.2)	575	(47.6)
3. If the self‐sampling tests are used for primary HPV screening, your willingness for testing HPV regularly will increase	14	(1.2)	99	(8.2)	579	(47.9)	518	(42.8)
4. Willingness level of using the self‐sampling kit again	2	(0.2)	45	(3.7)	735	(60.8)	427	(35.3)
5. Willingness level of introducing the self‐sampling kit to your relatives/friends	2	(0.2)	118	(9.8)	768	(63.5)	321	(26.6)
6. Easy‐to‐use level of using the self‐sampling kit to collect specimens	3	(0.2)	60	(5.0)	649	(53.8)	494	(41.0)
7. Safety feeling during the self‐sampling procedure	0	(0)	8	(0.7)	742	(61.4)	459	(38.0)
8. Comfort level after using the self‐sampling kit	0	(0)	91	(7.5)	845	(69.9)	273	(22.6)
9. User‐friendly level of the instructional manual for the self‐sampling kit	2	(0.2)	36	(3.0)	703	(58.1)	469	(38.8)

^a^ ITT, intention‐to‐treat. The questionnaire was originally written in Chinese and has been translated into English while maintaining the original intended meaning.

## 4 DISCUSSION

The participation rate of Taiwanese women aged 30–69 in cervical cancer screening, either through Pap test or HPV DNA test, has been consistently around 30% annually and 69% every 3 years.^20^ These tests have been provided free of charge by the government to women aged 30 and above since 1995. Despite the implementation of a national HPV vaccination program for schoolgirls aged 13–15 in 2018, the majority of women aged 40 and older have not been vaccinated against HPV. Consequently, it is anticipated that cervical cancer screening will remain essential for this demographic in the foreseeable future. However, there remains a significant proportion of women who do not engage in cervical cancer screening. Our study found that the likelihood of detecting CIN was higher among younger participants compared to older participants. Conversely, the incidence of cancer diagnosis was higher among older participants than younger ones. The data indicates that cervical cancer is most prevalent among women aged 25–45 in Taiwan. Younger women under 30 are less likely to undergo screening due to the out‐of‐pocket costs, potentially resulting in the detection of cervical lesions or cancer during their initial examination. Conversely, older women over 60 are at risk of being overlooked for cervical screening due to entrenched conservative beliefs, limited mobility, and the need for assistance from family members for medical appointments. While nearly 30% of women aged 30–50 have been screened, the rates drop to 17% for those aged 50–60 and 11% for those aged 60–70. To ensure a consistent comparison, the study included a higher number of participants with high HPV positivity rates. Notably, 34% of the participants exhibited cytological or histological HSIL, including cancer (inclusion criteria e). Consequently, statistical analysis reveals relatively high cancer rates across all age groups (4–12%), although this does not accurately reflect the true prevalence of cervical cancer in Taiwan. Generally, the progression from CIN to cancer takes approximately 10–20 years, providing a substantial window for detection and treatment. This underscores the importance of early screening and intervention in reducing the likelihood of cancer development.

The use of self‐collection of vaginal specimens for the detection of HPV DNA has been suggested as a more appealing option for engaging women who are difficult to reach in screening programs. Several published meta‐analyses have demonstrated the accuracy of HPV DNA testing on self‐collected specimens compared to those collected by physicians, using PCR‐based assays.^21^, ^22^, ^23^, ^24^ This practical alternative has the potential to increase participation in cervical cancer screening, particularly in low‐ and middle‐income countries where screening may not have been prioritized or where medical infrastructure is limited. Currently, there is a growing body of research focused on the use of urine as a medium for detecting HPV DNA, with several studies either underway or already commercialized.^25^, ^26^, ^27^, ^28^, ^29^, ^30^ While urine has been found to yield similar results to self‐collected vaginal specimens, some investigations have indicated that urine may be less sensitive than vaginal samples.^31^ This discrepancy has been attributed to the lower concentration of HPV DNA in urine compared to cervical samples, necessitating the development of a highly sensitive detection method to ensure ample HPV DNA detection.^25^, ^26^, ^27^, ^28^ Additionally, various factors such as storage conditions, DNA extraction and amplification methodology, and transportation have been shown to influence urine test results, leading to significant variability in clinical performance. However, a comparison of high‐risk HPV detections between urine and vaginal samples has revealed moderate consistency, suggesting a degree of similarity in HPV detection. Self‐sampling can help alleviate concerns related to embarrassment, discomfort, and anxiety. It is crucial to validate the collection device in conjunction with the specific PCR‐based HPV assay when using urine or vaginal cells as specimens for HPV testing. This validation is necessary to ensure the diagnostic effectiveness of the HPV test for self‐collected specimens. Furthermore, physicians may consider using HPV tests with a human internal control to assess the suitability of a sample, particularly when testing self‐collected specimens.

Our study confirmed our initial hypothesis that the HygeiaTouch Self Sampling Kit for Women is as reliable as physician‐sampled specimens for detecting HPV and diseases. It also performs similarly to a more standard nylon bristles device. The concordance between self‐collected and physician‐sampled specimens, evaluated using Cohen's kappa and target amplification method (PCR) with brush, swap, or tampon methods, ranged from 0.41 to 0.74. This is consistent with a meta‐analysis by Sy et al. that included 21 studies.^22^ In 2022, Arbyn et al. published a meta‐analysis comparing test agreement between HPV testing in self‐collected and physician‐sampled specimens from 26 studies.^23^ The positive agreement was 84.6%, negative agreement was 91.7%, and kappa was 0.72 for the pooled total agreement. Subgroup meta‐analyses showed that target amplification‐based DNA assays had higher overall agreement (90.4%) compared to signal amplification‐based DNA assays (86.7%) or RNA assays (82.3%). Our findings support previous research on the reliability of HPV testing using self‐collected specimens,^23^, ^24^ with substantial agreement (*κ* = 0.75 for HPV analyses) between self‐collected and physician‐sampled specimens. In our study, there were 140 discordant pairs of HPV positive results, with 71 specimens being negative according to the HygeiaTouch Self Sampling Kit for Women but positive according to physician‐sampled specimens, and 69 positive specimens by the HygeiaTouch Self Sampling Kit for Women yielding negative results in physician‐sampled specimens. The most significant reason for this discrepancy were confounding factors from the inclusion of participants who had ≥ HSIL and had undergone therapy. Additionally, the majority of these participants were HPV‐positive according to self‐collected specimens but negative according to physician‐sampled specimens. This is likely because self‐sampling primarily obtained vaginal cells, and vaginal specimens had a higher prevalence of noncarcinogenic HPV strains compared to cervical specimens.^32^ The type of α3/α15 phylogenetic species (HPV 61, 62, 71, 72, 81, 83, 84, and 89), which is roughly twice as prevalent in vaginal specimens as in cervical ones, is likely most responsible for this discrepancy. Our self‐sampling device is designed to gather cells exfoliated from the cervix by collecting cells from the vaginal area. This differs from the direct collection of cells from the cervix by physicians or other self‐collection devices, such as nasopharyngeal swabs, which may penetrate deeply near the cervix. This aspect represents a potential limitation of our self‐sampling device.

Arbyn et al. conducted a meta‐analysis which found that HPV tests employing self‐collected specimens yielded results comparable to those found when evaluating physician‐sampled specimens.^24^ The study aggregated data from 14 randomized controlled trials and reported that the sensitivity and specificity of HPV assays for detecting CIN2+ employing self‐collected specimens were 76% and 86%, respectively. In comparison, the sensitivity and specificity of HPV assays using physician‐sampled specimens for primary screening were 91% and 88%, respectively. Our findings indicated that the sensitivity, specificity, positive predictive value (PPV), and negative predictive value (NPV) when using vaginal cells collected using the HygeiaTouch Self Sampling Kit for Women for CIN2+ were highly similar to those when using cervical cells sampled by physicians, with a relative accuracy of 0.96.

Recent studies have consistently demonstrated the favorable reception of vaginal self‐sampling tools as a screening method among women.^33^, ^34^, ^35^ Women frequently cited ease of use, lack of shame, privacy, and comfort as reasons for choosing self‐sampling over physician sampling. Our findings align with previous research conducted across various populations, which also highlighted women's acceptance, experience, comfort, and preference. Women perceived self‐sampling as less unpleasant, more convenient, and less embarrassing compared to physician sampling, consistent with earlier studies. Privacy emerged as the primary benefit for choosing self‐sampling, provided that sufficient instruction on specimen collection was provided. Conversely, among those who opted for physician sampling, concerns about the accuracy of HPV self‐sampling results, doubts about their ability to properly self‐sample, and confidence in the established procedure of collecting Pap smears were the most influential factors.

A well‐designed self‐collection tool for gynecological purposes has the potential to effectively collect appropriate specimens while also providing women with a heightened sense of security and comfort. The HygeiaTouch Self Sampling Kit for Women has been engineered with a focus on product safety, as evidenced by its patented design (Patent No.: US D869,679S). The inclusion of a double‐layer structure prevents the collecting material from dislodging, and a petal stopper protects users from inserting the sampling device too far into the vaginal cavity. An anatomically accurate design ensures easy insertion and minimizes the risk of harm. Additionally, the kit allows for the simultaneous collection of sufficient cervical and upper vaginal cells. In our study, the majority of participants found the HygeiaTouch Self Sampling Kit for Women to be convenient and comfortable to use. It is important to note that nearly 90% of cervical cancer‐related deaths occur in low‐ and middle‐income countries, where barriers to completing screening procedures exist due to factors such as religious beliefs, social customs, and limited access to medical resources. The usability of the collection device plays a crucial role in enhancing women's acceptance and adherence to cervical cancer screening and HPV testing through self‐sampling methods in these regions. Furthermore, the samples obtained using this tool hold promise for the detection of various cancers affecting the female reproductive system, as well as for microbial research and the identification of sexually transmitted diseases.

## 5 CONCLUSIONS

This study demonstrated that the results of PCR HPV typing obtained from samples collected by women themselves using the “HygeiaTouch Self Sampling Kit for Women” are highly concordant with the results for samples obtained by physicians, as indicated by an overall kappa value of 0.75. Analysis of satisfaction questionnaires revealed that over 90% of participants expressed high levels of satisfaction with the kit's usability, and the incidence of adverse events was 0.7%, primarily consisting of mild vaginal pain or psychological factors. Self‐sampling for HPV testing is already implemented as a cervical screening option in certain countries. This trial further substantiates the notion that well‐designed self‐collection kits can offer a convenient, safe, and effective means of specimen collection, thereby enhancing women's willingness to undergo cervical screening.

## AUTHOR CONTRIBUTIONS

**Chung‐Yao Yang:** Data curation (equal); formal analysis (equal); methodology (equal); writing—original draft (equal) and writing—review & editing (equal). **Ting‐Chang Chang:** Conceptualization (lead); funding acquisition (lead); investigation (lead); methodology (equal); resources (lead); supervision (equal); writing—original draft (equal) and writing—review & editing (equal). **Hung‐Hsueh Chou:** Investigation (lead) and writing—review & editing (equal). **Angel Chao:** Investigation (equal) and writing—review & editing (equal). **Shih‐Tien Hsu:** Investigation (equal) and writing—review & editing (equal). **Yu‐Hsiang Shih:** Investigation (equal) and writing—review & editing (equal). **Huei‐Jean Huang:** Investigation (equal) and writing—review & editing (equal). **Cheng‐Tao Lin:** Investigation (equal) and writing—review & editing (equal). **Min‐Yu Chen:** Investigation (equal) and writing—review & editing (equal). **Lou Sun:** Investigation (equal) and writing—review & editing (equal). **Kuan‐Gen Huang:** Investigation (equal) and writing—review & editing (equal). **Kai‐Yun Wu:** Investigation (equal) and writing—review & editing (equal). **Wu‐Chiao Hsieh:** Investigation (equal) and writing—review & editing (equal). **Yi‐Ting Huang:** Investigation (equal) and writing—review & editing (equal). **Liang‐Hsuan Chen:** Investigation (equal) and writing—review & editing (equal). **Chien‐Hsing Lu:** Investigation (lead) and writing—review & editing (equal). **Hao Lin:** Investigation (lead); writing—review & editing (equal). **Chao‐Min Cheng:** Funding acquisition (equal); methodology (equal); resources (equal); writing—review & editing (equal).

## CONFLICT OF INTEREST STATEMENT

Chung‐Yao Yang is employed by Hygeia Touch Inc. in Taiwan. The remaining authors declare that the research was conducted in the absence of any commercial or financial relationships that could be construed as a potential conflict of interest.

### PEER REVIEW

The peer review history for this article is available at https://www.webofscience.com/api/gateway/wos/peer‐review/10.1002/btm2.10653.

## Data Availability

The data that support the findings of this study are available on request from the corresponding author. The data are not publicly available due to privacy or ethical restrictions.
